# Shaping retroviral genomes

**DOI:** 10.1186/1742-4690-8-S2-O2

**Published:** 2011-10-03

**Authors:** Andrew Lever

**Affiliations:** 1 University of Cambridge, UK

## 

We have been studying the structure and function of the 5' region of a number of retrovirus genomes. Using a combination of biochemical and biophysical techniques we have developed 2-dimensional and in some cases 3-dimensional structures for a number of lentivirus packaging signal regions including HIV-1 and FIV. The general features of these regions are of an unusually high level of sequence conservation reflecting the requirement for certain structures to be formed to allow transportation and processing of the viral RNA. There is in addition, data from our work and from that of others that the RNA structure is not fixed and that it changes on interaction with various ligands, including the viral proteins involved in genome RNA encapsidation. The model we are building up is one, in which, *cis*-acting sequences are able to adopt a variety of different structures to interact with viral, and probably cellular ligands to effect a highly divergent series of functions, as the viral RNA traffics through the cell from transcription to translation and encapsidation.

